# Hardness and Surface Roughness of 3D-Printed ASA Components Subjected to Acetone Vapor Treatment and Different Production Variables: A Multi-Estimation Work via Machine Learning and Deep Learning

**DOI:** 10.3390/polym17212881

**Published:** 2025-10-29

**Authors:** Çağın Bolat, Furkancan Demircan, İlker Gür, Bekir Yalçın, Ramazan Şener, Ali Ercetin

**Affiliations:** 1 Department of Mechanical Engineering, Faculty of Engineering and Natural Sciences, Samsun University, 55420 Samsun, Türkiye; 2 Department of Software Engineering, Faculty of Engineering and Natural Sciences, Samsun University, 55420 Samsun, Türkiye; furkancan.demircan@samsun.edu.tr (F.D.); ilker.gur@samsun.edu.tr (İ.G.); 3 Department of Mechanical Engineering, Faculty of Technology, Afyon Kocatepe University, 03200 Afyonkarahisar, Türkiye; bekiryalcin@aku.edu.tr; 4 Department of Marine Engineering, Maritime Faculty, Bandırma Onyedi Eylul University, 10200 Bandırma, Türkiye; rsener@bandirma.edu.tr; 5 Department of Naval Architecture and Marine Engineering, Maritime Faculty, Bandırma Onyedi Eylul University, 10200 Bandırma, Türkiye; aercetin@bandirma.edu.tr; 6 University College, Korea University, 145 Anam-ro, Seoul 02481, Republic of Korea

**Keywords:** acrylonitrile styrene acrylate, fused deposition modeling, Shore hardness, surface roughness, machine learning, deep learning

## Abstract

This paper analyzes the combined effects of acetone vapor treatment and 3D printing process parameters (layer thickness and infill rate) on the hardness and surface roughness of acrylonitrile styrene acrylate (ASA) components by using different machine learning and deep learning strategies for the first time in the technical literature. Considering the high-performance materials and aesthetic requirements of manufacturers, post-processing operations are highly critical for 3D-printed samples. ASA is a promising alternative, especially for the structural parts utilized in outdoor conditions like car outer components, electronic part housing, extreme sports equipment, and construction materials. However, it has to sustain hardness features against outer scratching, peeling, and indentations without losing its gloss. Together with the rising competitiveness in the search for a high-performance design with a perfect outer view, the combination of additive manufacturing and machine learning methods was implemented to enhance the hardness and surface quality properties for the first time in the literature. Concordantly, in this study, four different vaporizing durations (15, 45, 90, and 120 min.), three different layer thicknesses (0.1, 0.2, and 0.4 mm), and three different infill rates (25, 50, and 100%) were determined. According to both experimental and multi-way learning approaches, the results show that the support vector regressor (SVR) combined with one-dimensional convolutional neural networks (1D-CNNs) was the best approach for predictions. Gradient boosting (GB) and recurrent neural networks (RNNs) may also be preferable for low-error forecasting. Moreover, although there was a positive relationship between the layer thickness/infill rate and Shore D hardness outcomes, the highest levels were obtained at 45 min of vaporizing.

## 1. Introduction

Three-dimensional printing, also known as additive manufacturing, is a transformative technology that constructs objects layer by layer from digital models. Its ability to prototype and produce complex geometries offers significant advantages across various sectors. In healthcare, 3D printing enables the creation of patient-specific implants, prosthetics, and even bio-printed tissues, improving patient outcomes and reducing surgical risks [1,2,3]. In the automotive and aerospace industries, it supports lightweight part production, cutting material waste and enhancing fuel efficiency [4,5]. Architecture and construction benefit from its capacity to build intricate models and even full-scale structures faster and with fewer resources [6]. In education, 3D printing brings concepts to life, promoting hands-on learning in science, engineering, and design. Additionally, in the fashion and consumer goods sectors, it enables mass customization and rapid design iterations, catering to personalized preferences [7]. Overall, 3D printing enhances innovation, reduces costs, and shortens production times, positioning it as a game-changer across numerous industries.

According to the ISO 52900 standard (a detailed analysis appears in the Supplementary Materials), 3D printing technologies are categorized into seven main process types, each with unique advantages [8,9]. Material Extrusion, such as Fused Deposition Modeling (FDM), involves depositing melted material layer by layer. It is widely used for its low cost, simplicity, and suitability for prototyping and functional parts [10]. Vat Photopolymerization, including stereolithography (SLA), uses light to cure liquid resin in a vat. This method provides high-resolution and smooth surface finishes, ideal for detailed models like dental or jewelry applications [11]. Powder bed fusion (PBF), such as Selective Laser Sintering (SLS) or Direct Metal Laser Sintering (DMLS), uses a laser or electron beam to fuse powder materials. PBF excels in producing strong complex parts with excellent mechanical properties, particularly in aerospace and medical implants [12]. Material Jetting involves depositing droplets of material that solidify under UV light, offering high precision and multi-material printing, often used in prototypes requiring visual and functional realism [13]. Binder Jetting uses a liquid binder to fuse powder particles [14]. It is valued for its speed and low cost, especially in producing sand molds or metal parts for low- to mid-volume manufacturing. Sheet Lamination bonds layers of material sheets using adhesives or ultrasonic welding [15]. It is efficient for creating large objects quickly, often used in packaging or automotive mock-ups. Finally, Directed Energy Deposition (DED) involves focused thermal energy to melt materials as they are deposited. DED is particularly useful in repairing or adding features to existing parts, commonly seen in the aerospace and defense industries [16]. Each technique under ISO 52900 serves specific needs, enabling tailored solutions for various applications by balancing factors such as speed, material compatibility, cost, and mechanical performance.

Fused Deposition Modeling (FDM) is one of the most widely used 3D printing technologies due to its affordability, ease of use, and versatility. In FDM, thermoplastic filaments are heated and extruded through a nozzle, depositing material layer by layer to form the final object. This process makes FDM suitable for both prototyping and functional part production. The common thermoplastic materials used in FDM include PLA (Polylactic Acid), ABS (Acrylonitrile Butadiene Styrene), PETG (Polyethylene Terephthalate Glycol), TPU (Thermoplastic Polyurethane), and PA (Polyamide) [17,18]. PLA is one of the most popular choices due to its ease of printing, biodegradability, and low warping, making it ideal for aesthetic models and educational purposes [19]. ABS is stronger and more heat-resistant than PLA, making it suitable for functional parts, although it requires a heated bed and proper ventilation due to emissions [20]. PETG combines the strength and durability of ABS with the ease of printing of PLA, offering good impact resistance and minimal warping, which makes it ideal for mechanical parts and enclosures [21]. TPU is a flexible rubber-like material often used for producing gaskets, seals, or wearable items [22]. Nylon is a tough abrasion-resistant material suitable for high-stress applications, although it requires controlled humidity conditions due to its moisture sensitivity [23].

Acrylonitrile Styrene Acrylate (ASA) is an amorphous thermoplastic known for its excellent chemical resistance, UV stability, and weatherability, which make it highly suitable for outdoor applications [24]. Its chemical properties include good resistance to heat and oxidation, as well as a variety of chemicals, such as acids and bases, while maintaining toughness and impact strength comparable to ABS. ASA is widely used in automotive components, electrical housings, sports equipment, and construction materials due to its balance of durability, aesthetics, and resistance to discoloration under prolonged sunlight exposure. Its real-world applications include exterior automotive trims, outdoor furniture, roofing components, and protective housings for outdoor electronic devices. In additive manufacturing, particularly FDM-style 3D printing, ASA is valued as a functional engineering filament because of its high strength, dimensional stability, and superior weather resistance compared to ABS [25]. It is commonly used for prototyping and end-use parts that must endure outdoor conditions, such as signage, enclosures, and structural components. Additionally, ASA’s lower tendency to yellow under UV exposure and its smooth surface finish make it a preferred choice for both functional prototypes and aesthetic outdoor products.

In scholarly archives, FDM applications of ASA are often evaluated regarding how printing parameters such as raster angle, layer thickness, building direction, extrusion temperature, and infill density affect the resulting mechanical performance (tensile, flexural, and compression response), with many studies highlighting ASA’s ABS-like strength combined with improved outdoor stability [26,27]. Additionally, the impact resistance and high-speed deformation response of FDM-printed ASA were analyzed by different research teams [28]. In some studies, the vibration and natural frequency features of FDM-printed ASA were scrutinized, and numerical models were developed to estimate the first natural frequency values [29]. Convsersely, prominent features include its resistance to UV light and weathering, where researchers consistently report that ASA parts retain color and strength better than ABS when exposed to prolonged outdoor conditions [30]. Another active area of research focuses on reinforcing ASA with fibers, particularly carbon fibers, which significantly improve stiffness and strength, expanding its use in lightweight load-bearing applications [31]. Investigations into post-processing, such as thermal annealing, show that, while stress relief and dimensional stability can be achieved, distortion risks remain due to ASA’s amorphous nature [32]. Fracture mechanics and defect sensitivity, including the role of notches and interlayer adhesion, are also widely analyzed to better predict failure behavior in functional designs [33]. Beyond property optimization, application-driven studies demonstrate ASA’s effectiveness in printed molds, housings, and outdoor enclosures, validating its suitability for long-term exposure to UV and moisture.

Contrary to the existing literature, this paper addresses the secondary post-processing opportunities for FDM-printed ASA components by way of an additional vaporizing treatment immediately after the primary production stage. Thanks to the vaporizing treatment, there is a valuable engineering solution for better surface finishings and optimized hardness levels in ASA parts. In this paper, the integrated influences of the 3D printing variables (layer thickness and infill rate) and post-evaporating treatment (vaporizing time in acetone environment) were investigated by using both experimental and machine learning approaches for the first time in the literature specifically for 3D-printed ASA. To achieve the most accurate predictive model, different machine learning, deep learning, and hybrid models were implemented regarding the fabricated ASA samples to obtain accurate final hardness and surface roughness values. This study aims to establish a reliable predictive framework for modeling the hardness and surface roughness of ASA components, serving as a bridge between practical 3D printing applications and novel machine learning methodologies.

## 2. Materials and Methods

### 2.1. Utilized Thermoplastic Material, 3D Printing Stage, and Vaporizing Procedure

In this work, ASA filament was selected as a target material owing to its high resistance against UV radiation, perfect weathering endurance, sufficient fluidity for the FDM process, superior color stability, and successful impact responses. In this context, the ASA filament used in this study was supplied by Elas 3D Filament and Plastic Machinery Co., Ltd. (Kocaeli, Türkiye). According to the supplier’s recommendations for FDM processing, the material was printed with a suitable bed and nozzle temperatures considering the glass transition temperature of the ASA. Table 1 indicates all the details of the utilized filament material.

**Figure 1:**
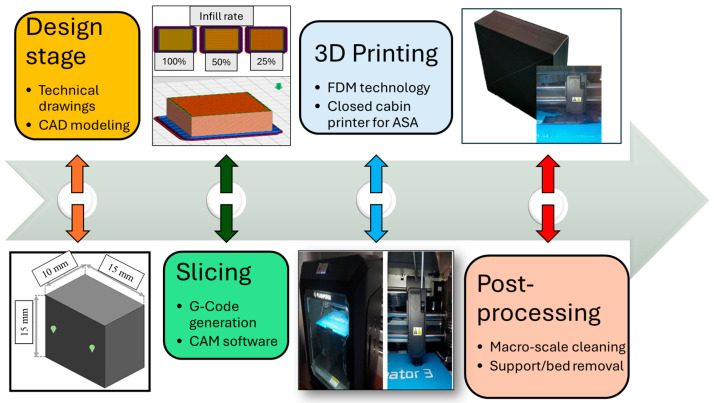
Main process flow of the FDM efforts.

To evaluate the mechanical performance and surface quality levels of the fabricated specimens, 3D solid models of the hardness test specimens were designed in SolidWorks 2020^®^. Repetitive hardness tests were conducted in accordance with ASTM D2240-15 standard, and the Shore D hardness durometer (Loyka LX-D model) was utilized in the measurements. According to this criterion, the test sample thickness and width values should be at least 6 mm and 12 mm, respectively. Also, a minimum distance of 6 mm is required between the measurement points. Therefore, hardness test samples with dimensions of 15 × 15 × 10 mm^3^ were manufactured by applying the proper FDM process parameters. Accordingly, the created 3D model was exported from the SolidWorks part format (.sldprt) to a stereolithography file (.stl) and subsequently processed in Flashprint 5.0 (Flashforge). The slicing software generated G-code files corresponding to related infill rates and layer thickness levels. The real fabrication efforts were realized with a Flashforge Creator 3 dual-extruder 3D printer (Jinhua, China). The enclosed building chamber of the printer allows for control of airflow and ambient humidity. The system enables a maximum printing speed of 150 mm/s, with adjustable parameters for travel, first layer, outer layers, and overall print speed. Printing was performed with a 0.4 mm hardened nozzle featuring self-heating capable of reaching 300 °C, while the flexible magnetic build plate reached up to 120 °C and provided a working volume of 200 × 250 × 300 mm^3^. Figure 1 illustrates the process flow of the FDM efforts.

**Figure 2:**
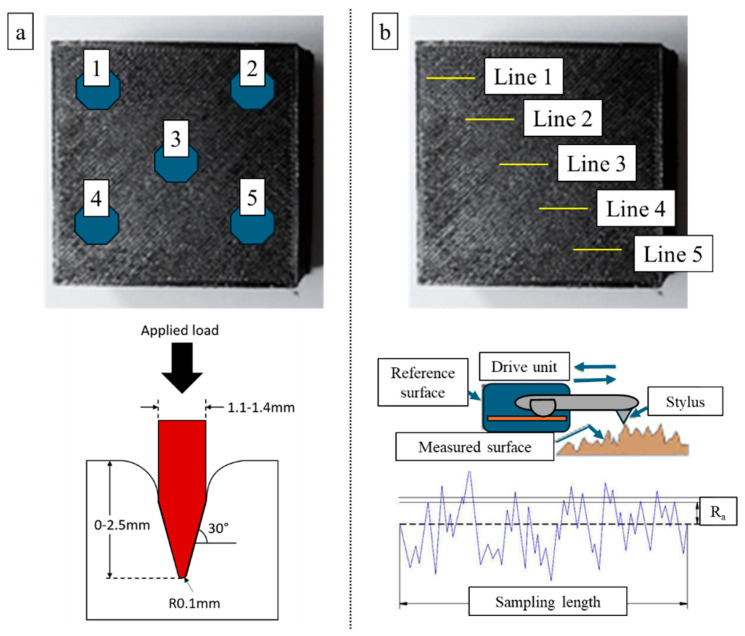
Measurement points in hardness (**a**) and surface roughness (**b**) evaluations.

To measure the surface roughness results, a surface profilometer setup (Mahr MarSurf PS1 model; Göttingen, Germany) was used, and R_a_ results were adopted in the comparisons. Measurements were carried out at room temperature. When each pass was completed, the surface was cleaned and re-measured with the contact profilometer to obtain the corresponding roughness. Five measurements for workpiece surface roughness were carried out and averaged for each pass. The cutting length (L_c_) was chosen as 0.8 mm and the sampling length (L_t_) as 4.8 mm for the measurement of the surface roughness values. Figure 2 demonstrates the measuring details and data collection strategy for both surface roughness and hardness evaluations.

**Figure 3:**
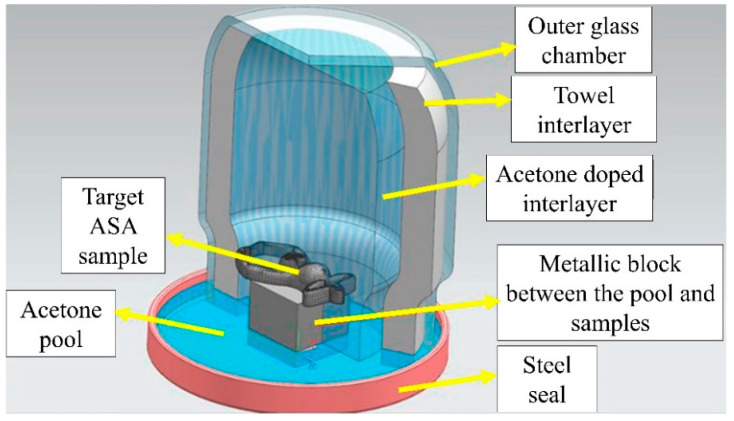
Schematic view of the vaporizing environment and equipment.

In the setup shown in Figure 3, the outer glass chamber was sealed with a steel lid to block the liquid and gas leakage through mechanical montage. A metallic block was placed at the center of the bottom steel lid to hinder direct contact between the liquid acetone and the target ASA samples. Thanks to its rapid delivery from the domestic suppliers, cost-effectiveness, and suitable surface interaction features, acetone was selected in this work even though dimethylformamide (DMF) and dichloromethane were also tried for acrylonitrile-based polymers. The outer glass chamber was covered with an acetone-doped towel, and an extra interlayer was created to feed the acetone gas environment with additional vapor, thereby providing better gas uniformity in the chamber. The vapor chamber volume was 400 cm^3^, and a total of 30 mL of acetone was used in the chamber. Also, all treatments were conducted at room temperature, and the macro-scale shape transformations were monitored with glass outer frames. At the bottom side of the chamber, an acetone pool was formed to compound the upward vapor delivery. Following the installation of the vaporizing treatment setup, four different application durations were appointed as 15, 45, 90, and 120 min. For each test, a new vapor environment was built in order to attain identical conditions. Moreover, together with vaporizing, all input variables and their levels in this work can be monitored in Table 2.

### 2.2. Machine Learning (ML) and Deep Learning (DL) Details

This study applied a broad set of machine learning (ML) and deep learning (DL) models to predict hardness and surface roughness from additive manufacturing process data, focusing on algorithms with distinct strengths for handling nonlinear multi-target problems. Gradient boosting (GB) [34] and its optimized variant XGBoost [35] leveraged sequential error correction and regularization to efficiently capture complex parameter–property relationships. Ensemble tree methods such as Random Forest (RF) [36], Extra Trees (ETs) [37], and bagging [38] enhanced robustness to noise through randomized feature and sample selection, while stacking [39] integrated multiple base models to exploit complementary predictive capabilities. AdaBoost [40] builds an ensemble of weak learners sequentially, where each new model places greater weight on previously misclassified samples. This iterative reweighting strategy often improves predictive accuracy compared to a single base learner. However, because AdaBoost continues to emphasize all misclassified points, it can overfit when the dataset contains noisy or mislabeled samples, leading to reduced generalization performance.

DL approaches were included for their ability to learn representations from raw or minimally processed inputs. Multi-Layer Perceptrons [41] (MLPs) modeled general nonlinear relationships, while one-dimensional convolutional neural networks [42] (1D-CNNs) identified local dependencies in sequential parameter data and two-dimensional convolutional neural networks (2D-CNNs) processed structured grid-like input formats. Recurrent neural networks (RNNs) and Long Short-Term Memory [43] (LSTM) were incorporated to capture temporal dependencies across sequential layers, with LSTMs improving long-term memory retention. Traditional regression and proximity-based methods provided interpretable baselines. Support vector regressor [44] (SVR) demonstrated strong performance in nonlinear regression tasks, achieving the best performance when combined with 1D-CNN feature extraction. Decision Trees [45] (DTs) offered interpretability but were less robust alone, while K-Nearest Neighbor [46] (KNN) exploited parameter similarity for predictions. Ridge [47] and Bayesian Ridge [48] regression added regularization and uncertainty estimation, and linear regression [49] (LR) served as a simple reference point.

From a technical perspective, the chosen algorithms operate on distinct learning principles that influence their suitability for nonlinear multi-target regression tasks. GB and XGBoost construct additive models in a forward stage-wise fashion, where each successive tree is trained to minimize the residual errors of its predecessors, incorporating shrinkage (learning rate) and regularization terms to improve generalization. RF and ET utilize bootstrap aggregation (bagging) to train multiple DTs on random feature subsets, with RF using optimal splits and ETs using randomized split points to increase diversity among trees. Bagging reduces variance by averaging predictions from multiple base learners, while stacking integrates heterogeneous models through a meta-learner that is trained on their outputs. AdaBoost adapts to model weaknesses by iteratively reweighting misclassified or poorly predicted samples, focusing the learning process on more complicated cases.

DL models, in contrast, learn hierarchical feature abstractions through multiple layers of nonlinear transformations. MLPs consist of fully connected layers that map input features to outputs via learned weight matrices and activation functions, enabling flexible nonlinear modeling. CNNs apply learnable convolutional kernels to detect local patterns; in 1D-CNNs, these patterns occur along a single dimension (e.g., sequential process parameters), whereas in 2D-CNNs they operate across two-dimensional grids (e.g., spatially organized data). RNNs capture temporal or sequential dependencies by maintaining hidden states across time steps, with LSTMs enhancing this capability via gated mechanisms that regulate information flow and retain long-term dependencies. SVR maps inputs into high-dimensional feature spaces using kernel functions and constructs an optimal regression hyperplane within a defined margin of tolerance (ϵ), making it well-suited for smooth nonlinear predictions. KNN bases its predictions on the average (or weighted average) of the nearest neighbors in feature space, while Ridge and Bayesian Ridge regression introduce $l_{2}$-norm penalties to the loss function to control coefficient magnitudes, with Bayesian Ridge additionally modeling the regression problem probabilistically to account for uncertainty. LR, as the simplest baseline, assumes a purely linear relationship between inputs and targets without regularization, serving as a reference for performance gains achieved by more complex models.

In this study, a key enhancement was the integration of 1D-CNN-based feature extraction before regression, which reduced data dimensionality and transformed high-variance manufacturing parameters into compact discriminative representations. This hybrid approach substantially improved anticipation accuracy and generalization, thereby enabling simultaneous estimation of hardness and roughness without additional sensing hardware specific to this study.

### 2.3. Performance Metrics

In the present study, the predictive capabilities of the developed ML and DL models were systematically assessed using three complementary statistical metrics: the coefficient of determination ($R^{2}$), mean squared error (*MSE*), and root mean squared error (RMSE). These metrics were selected to provide a comprehensive evaluation of model performance in terms of accuracy, error magnitude, and generalization capability for both hardness and surface roughness prediction. The $R^{2}$, defined in Equation (1), quantifies the proportion of variance in the target variable that is explained by the model.

(1) $$R^{2}=1-\frac{\sum_{i=1}^{n}{\left(t_{i}-p_{i}\right)}^{2}}{\sum_{i=1}^{n}{\left(t_{i}-a_{i}\right)}^{2}}$$

Here, $t_{i}$ denotes the actual target value, $p_{i}$ the predicted value, $a_{i}$ the mean of the actual values, and n the total number of samples. While $R^{2}$ provides an overall measure of quality of fit, it does not reflect the absolute magnitude of prediction errors. To address this, the *MSE* was computed, as given in Equation (2):

(2) $$MSE=\frac{1}{n}\sum_{i=1}^{n}{\left(t_{i}-p_{i}\right)}^{2}$$

*MSE* penalizes larger deviations more severely, making it highly sensitive to significant prediction errors caused by variations in process parameters or measurement noise. To express the error in the same physical units as the measured properties, the *RMSE* was employed, as shown in Equation (3):

(3) $$RMSE=\sqrt{\frac{1}{n}\sum_{i=1}^{n}{\left(t_{i}-p_{i}\right)}^{2}}$$

*RMSE* allows for a direct comparison of prediction errors to tolerances that are acceptable in industrial applications. This provides a practical perspective on the suitability of models for deployment in manufacturing environments. All performance metrics were calculated using a five-fold cross-validation procedure. The dataset was randomly partitioned into five equal subsets. In each iteration, four subsets were used for training, and one subset was used for testing. The final performance scores represent the average of all folds, which minimizes bias due to data partitioning and ensures a robust evaluation of the entire dataset.

## 3. Results and Discussion

### 3.1. Experimental Analyses

**Figure 4:**
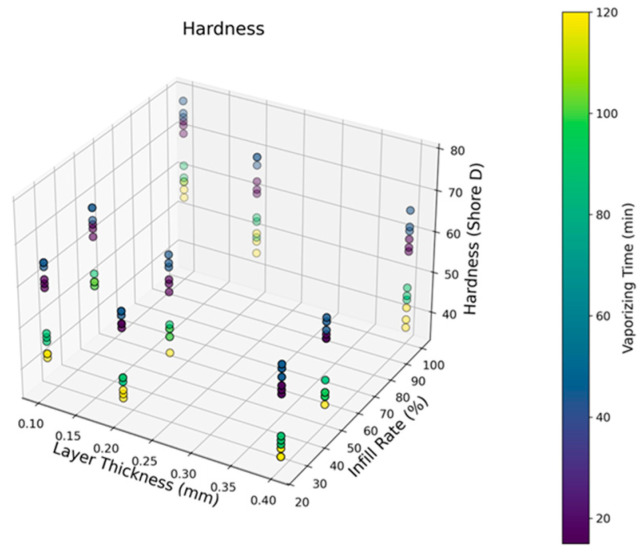
Hardness results depending on the layer thickness, infill rates, and vaporizing time.

Figure 4 demonstrates the Shore D hardness scores of the vapor-treated ASA samples depending on the shifting infill rates and layer thicknesses. From the outcomes, the highest average hardness value of 75.6 Shore D (with a standard deviation (SD) of 2.08) was detected for the 100% filled samples subjected to 45 min vaporizing treatment, whereas the lowest average hardness of 36.6 Shore D (SD: 1.15) belonged to the 20% filled samples subjected to 120 min vaporizing. Up to 45 min, a positive effect of the acetone was observed due to its polarized molecular structure, which triggers the reaction rate and improves bond structures [50]. However, with a rising vapor treatment duration, chain relaxation mechanisms emerge, and localized fusion rates gain non-uniform characteristics. Based on the complete dataset, no consistent increasing or decreasing trend was observed between the parameter sets and the measured hardness values. At this point, it can be observed that there is a turning point for a 45-min vaporizing time, and the measured hardness values start to drop after this time due to the ascending softening effect of gas treatment. Initially, the ASA samples have several peak and valley structures on their surfaces that may cause relatively large cavities on the test surfaces. However, after up to 45 min of vaporizing time, those kinds of cavities become smaller, and surface uniformity increases. This phenomenon is the probable reason for the hardness improvement in 45 min of vaporizing. On the other hand, rising layer thickness levels triggered higher hardness results for all the samples. This case can be explained by the escalating stacking layer amount due to its strong stacking ability against the converse loading forces. As for the infill rate, it can be proposed that, as the load-carrying polymer strut volume increases, the average hardness results increase to the peak levels. However, since vaporizing is effective solely on the limited volume of the part surface, the influence of the infill rate cannot be qualified as linear.

**Figure 5:**
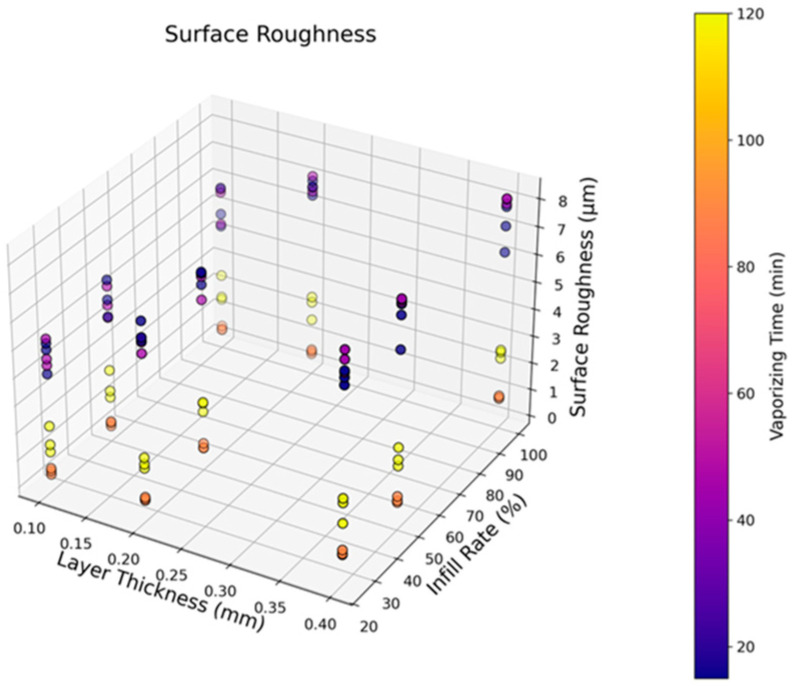
Surface roughness results depending on the layer thickness, infill rates, and vaporizing time.

Figure 5 shows the roughness values of the tested ASA samples according to altering the infill rate, layer thickness, and vaporizing time. The results show that there was a turning point in the 90-min vaporizing time, and this duration is the best option for the most successful surface quality. For the 50% filled samples, the lowest average roughness of 0.36 µm (SD: 0.08) was recorded, and the layer thickness was 0.1 mm. The highest average roughness of 8.08 µm (SD: 0.1) was detected for the sample with a 0.4 mm layer thickness and 45 min of vaporizing. This observation can be attributed to the optimized fusion of interlayers modified by consistent exposure to acetone vapor, thereby stimulating robust cohesion between the printing layers [51]. Also, the interlayer diffusion rate reaches a balanced level after 90 min of vaporization, localized corrugated sections decrease, and surface tops and valleys are minimized with steady-state fusion. With rising vaporizing time, the smoothing capacity of the acetone gas increases, and the diffusion ability on the sample surfaces enhances the atomic activity in the vicinity of rough cavities. Nevertheless, with more vaporizing time, excess softening effects were seen, and surface flows with unwanted waviness were present on the ASA parts. As the vaporizing duration reached 120 min, although the average roughness was still lower than the initial measurements, the highest-quality surfaces were obtained in the optimum conditions at 90 min. Additionally, lower layer thickness levels were advantageous for better surface view because of the closely packed production layers, causing smaller printing gaps to be closed by the gas treatment.

### 3.2. Machine Learning and Deep Learning Evaluations

The experiments were conducted on a high-performance desktop computer with a processor (Intel Core i9-13900K, Intel, Santa Clara, CA, USA) and dedicated GPU (NVIDIA RTX 4080, Nvidia, Santa Clara, CA, USA). All models were implemented in the Python (3.12) programming environment. ML models were developed using the scikit-learn library, and CNN-based models were implemented using the TensorFlow framework.

The dataset consisted of 108 experimental observations according to average values and is shared in Table 3. Three independent input variables were used: layer thickness, vaporizing time, and infill rate. The prediction targets were two continuous output variables: hardness and surface roughness.

**Figure 6:**
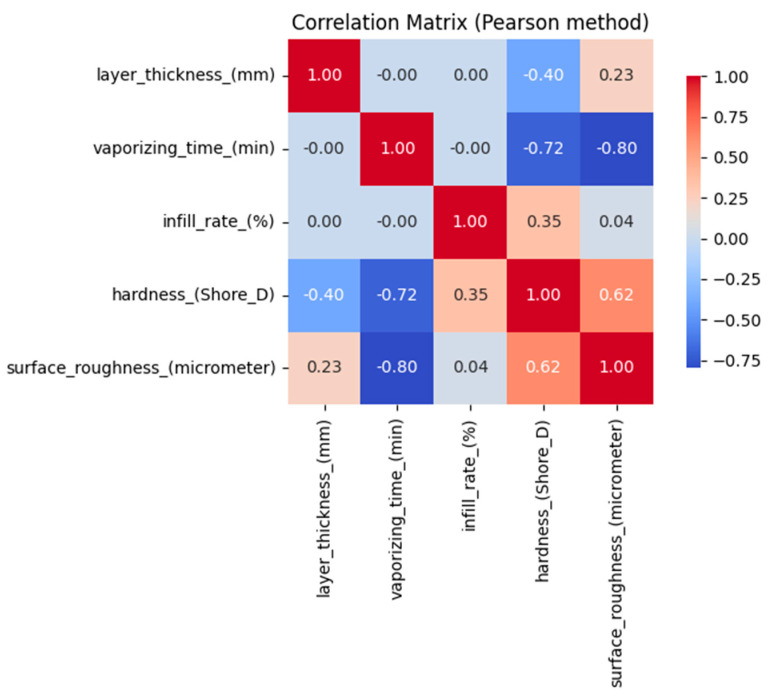
Correlation matrix of the dataset.

Three independent variables, layer thickness (mm), vaporizing time (min), and infill rate (%), were selected based on their known influence on the mechanical and surface characteristics of additively manufactured materials. The dependent variables were defined as hardness (Shore D) and surface roughness (µm), representing key performance indicators of the fabricated parts. The selection of these parameters was initially guided by process physics and previous studies, which report that layer thickness, post-processing duration, and infill density significantly affect material compaction and surface morphology. Correlation analyses were performed using the Pearson method to statistically validate the variable selection. As illustrated in Figure 6, the results revealed strong negative correlations between vaporizing time and both hardness (r = −0.72) and surface roughness (r = −0.80), indicating that prolonged vaporization decreases hardness and improves surface quality. Moderate relationships were also found between layer thickness and hardness (r = −0.40), and between infill rate and hardness (r = +0.35). Furthermore, the low intercorrelation among the input variables (|r| < 0.1) confirmed the absence of multicollinearity, ensuring that each input contributes independent information to the model. These findings collectively validate that the chosen independent and dependent variables are both physically meaningful and statistically justified for the regression framework.

To evaluate the model performance, the dataset was partitioned into training and testing subsets using a five-fold cross-validation strategy. In each fold, 86 samples (approximately 80%) were used for training the model, and the remaining 22 samples (approximately 20%) were reserved for testing. This ensured that each data point was included in both the training and testing phases across different folds, thereby improving the reliability and generalizability of the evaluation results.

Both the ML- and CNN-based models had a three-input, two-output architecture. In this configuration, the three process parameters (layer thickness, steaming time, and occupancy rate) were simultaneously provided as inputs to the model. These features were processed in parallel or joint feature-extraction pathways, enabling the models to capture the relationships and potential interactions between the parameters. The models then produced two separate predictions, one corresponding to hardness and the other to surface roughness, enabling simultaneous multi-target regression. Table 4 presents the detailed configurations, hyperparameters, and architectural parameters of the models used in this study.

In addition to the main experimental setup, 1D-CNNs were used to extract features. A total of 32 representative features were extracted from the three input parameters, resulting in a feature map of size 32. This feature map was then used as input for the regression models. Incorporating the 1D-CNN feature extraction stage enabled the models to capture local patterns and parameter-specific variations present in the data. This approach allowed the regression models to focus on these localized features during training and testing, enhancing their ability to model complex relationships between process parameters and target variables. Table 5 provides the detailed architecture and parameter configurations of the feature extraction model and regression heads.

The experimental study was designed to yield comprehensive and wide-ranging results by employing a diverse set of models, enabling a more thorough evaluation of predictive performance. Furthermore, combining ML and DL approaches allowed the study to leverage the strengths of both paradigms. This integration enhanced the robustness and capability of the modeling framework, improving its ability to capture linear and nonlinear relationships within the data and increasing the effectiveness and reliability of the results.

Table 6 summarizes the predictive performance of all the tested models for hardness and surface roughness, as determined by 5-fold cross-validation. The top-performing group consisted of CNN-based feature extraction combined with ensemble regressors. The best models were feature extraction with SVR (average MSE = 2.0941; average $R^{2}$ = 0.9614) and GB (average MSE = 2.2007; average $R^{2}$ = 0.958). Standalone DL models, such as 1D-CNN and 2D-CNN, achieved competitive accuracy. Notably, 1D-CNN slightly outperformed 2D-CNN, suggesting that one-dimensional convolutions are more effective at capturing structured relationships among the three process parameters. Traditional ensemble methods, including RF, bagging, and XGBoost, formed the second tier of performance, demonstrating robust, although slightly lower, accuracy. Purely linear models, such as Ridge, Bayesian Ridge, and linear regression, demonstrated poor predictive performance because they assume a strictly linear relationship between the input parameters and target variables. In this study, however, the relationships between process parameters (layer thickness, steaming time, and occupancy rate) and output properties (hardness and surface roughness) are inherently nonlinear and influenced by complex interactions. Linear models are unable to capture these dependencies, which leads to underfitting and considerably reduced accuracy, with average R^2^ values around 0.69.

By contrast, combining Ridge regression with CNN-based feature extraction (feature extraction with Ridge) markedly improved performance (avg R^2^ = 0.9556 compared with 0.6942 for standard Ridge). This enhancement arises because the 1D-CNN feature extractor transforms the three raw inputs into thirty-two higher-level features that encode nonlinear relationships and localized patterns. Projecting the data into this enriched feature space reshapes the problem so that the relationships between features and outputs become closer to linear. In this transformed domain, Ridge regression can operate effectively, applying regularization to prevent overfitting while exploiting the structure already embedded in the extracted features. The observed performance gain, therefore, highlights that the limitation of Ridge lies not in its optimization mechanism but in the inadequacy of raw inputs. Once the inputs are expressed through CNN-derived representations, even a simple linear model can achieve predictive accuracy comparable to more sophisticated algorithms. The fold-level performance of the top five models is provided in Table 7.

**Figure 7:**
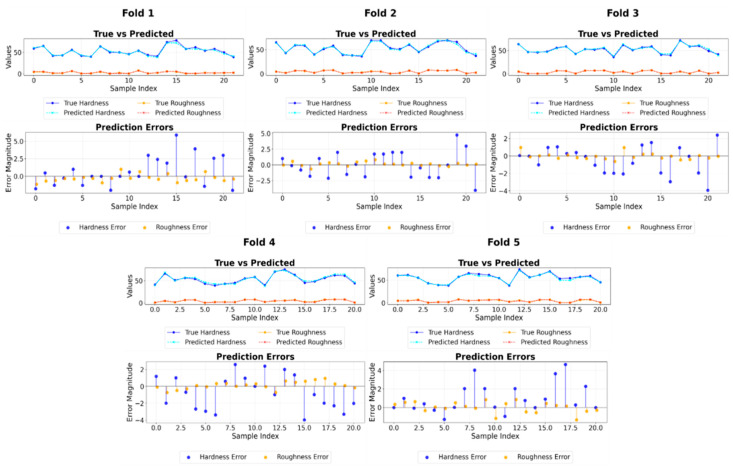
Fold-by-fold prediction performance of the feature extraction with the SVR model.

A fold-level analysis of the top five models (see Table 7) confirmed that they all maintained $R^{2}$ values above 0.94 across folds, indicating consistent predictive capability. Feature extraction with SVR displayed slightly higher variability in MSE between folds, likely due to changes in target value distributions. In contrast, 1D-CNN and GB exhibited strong stability in certain folds with notably low errors. Figure 7 visually supports these findings, showing that the predicted values closely track the actual measurements across all folds with minimal deviation.

**Figure 8:**
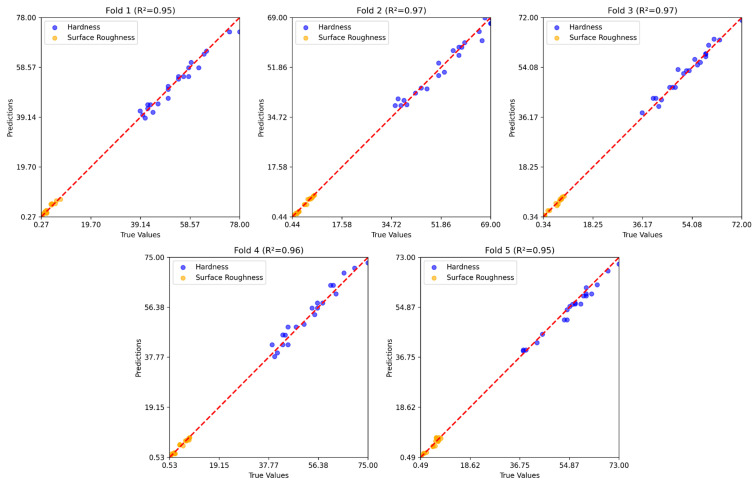
Five-fold regression results of the feature extraction with the SVR model.

**Figure 9:**
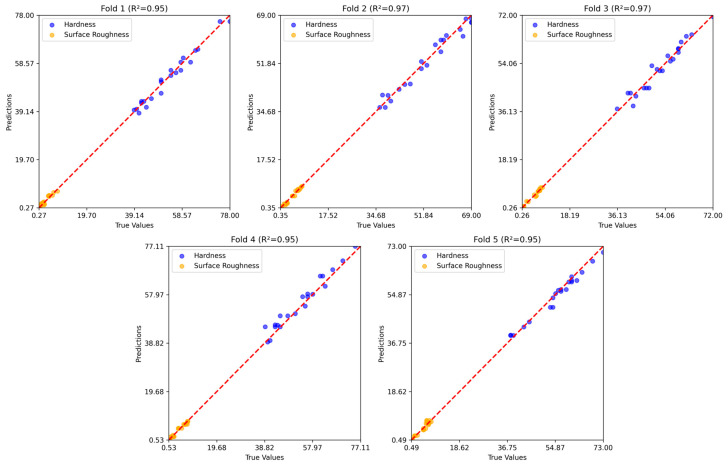
Five-fold regression results of the GB model.

**Figure 10:**
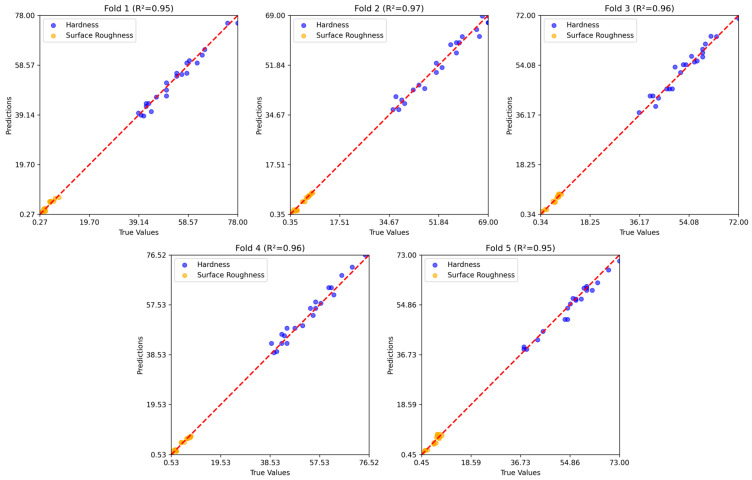
Five-fold regression results of the RNN model.

Figure 8, Figure 9 and Figure 10 show the five-fold regression results of the feature extraction with SVR, GB, and RNN models for predicting hardness and surface roughness. In all cases, the predicted values align closely with the true values along the 1:1 reference line, confirming strong predictive accuracy. The feature extraction with the SVR model achieved the most consistent performance, with $R^{2}$ values ranging from 0.95 to 0.97, demonstrating its ability to generalize effectively across data partitions. Gradient boosting also produced robust results, closely tracking the true values across folds, although slight variability in prediction error indicates some sensitivity to the composition of training and testing subsets. The RNN model maintained high predictive accuracy, with $R^{2}$ values consistently above 0.94, but exhibited marginally greater dispersion around the reference line compared to the other two models.

**Figure 11:**
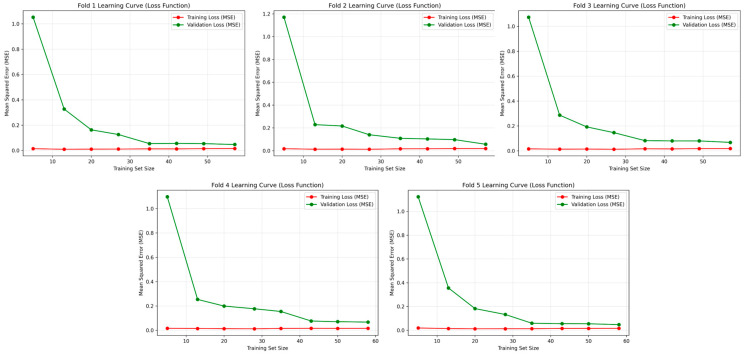
Learning curves of the best-performing CNN–SVR model (training and validation losses) across five folds of cross-validation.

To assess dataset adequacy and validate model generalization given the limited sample size (108 observations), learning curves were generated for the superior CNN–SVR model using five-fold cross-validation, as illustrated in Figure 11. Each subplot depicts the evolution of training and validation mean squared error (MSE) as a function of increasing training set size. The learning dynamics reveal a characteristic pattern across all folds: both training and validation curves exhibit rapid error reduction during the initial training phase, subsequently achieving smooth convergence toward lower MSE values. The close correspondence between training and validation trajectories throughout the learning process provides compelling evidence of effective generalization without overfitting. Notably, the absence of divergence between these curves demonstrates that the available dataset contains sufficient informational content to enable the CNN–SVR model to establish stable and representative mappings between process parameters and material properties. Furthermore, the convergence plateau observed beyond approximately 40–50 training samples indicates that the model approaches its optimal learning capacity within the limitations of the current dataset, suggesting that substantial data augmentation would yield only incremental performance gains.

**Figure 12:**
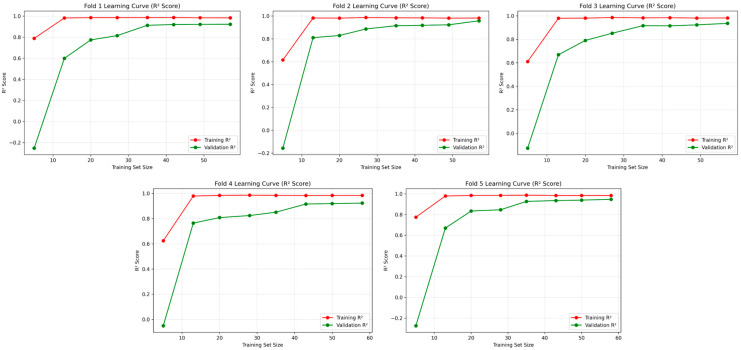
Learning curves of the best-performing CNN–SVR model (R^2^ score) across five folds of cross-validation.

To provide complementary insights into model learning behavior, Figure 12 presents the coefficient of determination (R^2^) evolution for the CNN–SVR model across all cross-validation folds. Each subplot illustrates the progressive development of training and validation R^2^ scores with increasing sample size, offering additional perspective on generalization capability. The learning curves consistently demonstrate an upward trajectory across all folds, with validation R^2^ values steadily converging toward their training counterparts as data availability increases. This convergence pattern substantiates effective learning of the underlying input–output relationships without manifestations of underfitting or overfitting phenomena. The stabilization of both curves at elevated R^2^ levels (consistently exceeding 0.9) across all validation folds confirms robust predictive performance and reliable generalization despite dataset size limitations. These findings collectively validate that the CNN–SVR architecture successfully captures the complex nonlinear interdependencies among process parameters (layer thickness, infill rate, and vaporizing time), thereby enabling precise simultaneous prediction of surface roughness and hardness in ASA-based FDM components.

## 4. Discussion

ML and DL techniques have attracted considerable attention due to their ability to predict mechanical and surface properties in additive manufacturing processes. While many studies have focused on individual properties, such as hardness or surface roughness, these studies collectively demonstrate the potential of advanced algorithms to capture complex nonlinear relationships between manufacturing parameters and material performance. In this context, ensemble methods, as well as various neural network architectures, have repeatedly emerged as leading approaches. A summary of the studies conducted in this field to date is presented in Table 8.

For instance, Veeman et al. (2023) [54] obtained an $R^{2}$ value of 0.9136 for ABS component hardness prediction using RF, and Mahmoud et al. (2024) [56] reported an $R^{2}$ of 0.995 for PC parts using the same model. Similarly, Badogu et al. (2024) [55] obtained an $R^{2}$ of 0.955 for PLA hardness prediction using ensemble learning, which reinforces RF’s strong generalization capability across polymer-based additive manufacturing applications. Similar trends have been observed in research on surface roughness prediction, although the approaches often differ in feature sources and computational strategies. Tzotzis et al. (2025) [63] achieved a minimum mean absolute percentage error (MAPE) of 1.51% for carbon fiber-reinforced polymer (CFRP) machining by integrating vibration-derived features into artificial neural network (ANN) models. Wardhani et al. (2024) [57] employed hybrid regression techniques and reported RMSE values between 3.21 and 3.65 for surface roughness estimation. In the field of metal additive manufacturing, Panico et al. (2025) [61] applied CNN-based models to predict the mechanical properties of laser powder bed fusion, achieving an $R^{2}$ of approximately 0.94. Meanwhile, Mulugumdam et al. (2025) [59] employed XGBoost to model a single quality metric in fused deposition modeling (FDM), achieving an MSE of 0.799. While these studies demonstrate the feasibility of achieving high predictive accuracy, most prior efforts are limited to single-property modeling or depend on auxiliary sensing systems. Although these systems are effective, they increase the complexity and cost of implementation.

Unlike previous studies with a single-target scope, the present study develops a multi-target regression framework that can predict hardness and surface roughness simultaneously from process parameter data alone. The proposed model, which combines one-dimensional CNN-based feature extraction with SVR, achieved an average MSE of 2.0941 and an $R^{2}$ of 0.9614 across five-fold cross-validation. These results surpass the performance reported by Veeman et al. (2023) [54] by approximately 5.2% in $R^{2}$ and marginally exceed the accuracy of Badogu et al. (2024) [55] despite addressing a more complex prediction problem. Additionally, the framework matches Tzotzis et al.’s (2025) [63] performance in surface roughness prediction without requiring vibration-sensing hardware, thus simplifying its integration into industrial workflows. Reducing RMSE to approximately 1.45 significantly improves upon the surface roughness results reported by Wardhani et al. (2024) [57].

The superior performance of the proposed CNN–SVR hybrid can be attributed to its ability to combine the nonlinear feature extraction capacity of convolutional neural networks with the generalization stability of support vector regression. The CNN layer captures complex localized relationships between layer thickness, infill rate, and vaporizing time—relationships that directly reflect the physical interactions affecting surface finish and hardness in FDM. The SVR component then operates on these high-level features within a compact and less redundant representation space, resulting in enhanced regression accuracy and reduced overfitting.

Several factors contribute to the overall strength of the proposed methodology. The framework demonstrates versatility by effectively integrating both machine learning and deep learning paradigms, allowing the advantages of each to be leveraged in a complementary manner. It has been comprehensively benchmarked against a broad range of algorithms, ensuring a rigorous and balanced assessment of its relative performance. Moreover, the proposed model achieves high predictive accuracy without relying on costly or complex measurement systems, making it particularly suitable for rapid, low-cost, and multi-criteria quality evaluation in manufacturing environments. Despite these strengths, certain limitations remain. Although the manually designed feature extraction stage proved to be effective, it could benefit from automated optimization or dimensionality reduction techniques to further enhance adaptability and scalability. In addition, while the dataset used in this study was sufficient for the intended analysis, it was restricted to a single material type and a relatively narrow range of process parameters. Expanding the dataset to include multiple materials, part geometries, and processing conditions would likely improve the model’s generalization and robustness. Unlike many existing models that depend on extensive sensing data, the proposed framework achieves reliable predictive performance using only process parameters and post-processing conditions, underscoring its practicality for low-cost real-time quality prediction in industrial additive manufacturing applications.

Future work could further enhance prediction accuracy by integrating the representational learning capabilities of CNNs with the stability of ensemble methods in hybrid architectures. Incorporating metaheuristic optimization algorithms, such as genetic algorithms or particle swarm optimization, may improve hyperparameter tuning and model efficiency. Furthermore, using explainable AI tools, such as SHAPs or LIMEs, would provide transparency in model decision-making and yield more profound insights into process–property relationships. This would ultimately support more effective process optimization in industrial additive manufacturing.

## 5. Conclusions

This study focused on applying ML and DL techniques to simultaneously predict hardness and surface roughness in additively manufactured ASA components. The following key conclusions can be drawn from the outcomes of this study:

- The proposed ML–DL framework, which integrates 1D-CNN feature extraction with SVR, was highly effective in modeling the complex nonlinear relationships between process parameters and output properties.
- This approach successfully predicted two critical quality indicators, hardness and surface roughness, simultaneously, eliminating the need for separate models and additional sensing hardware.
- Among all the tested algorithms, the 1D-CNN model with SVR had the best overall performance, with an average R^2^ of 0.9614 and an MSE of 2.0941 across five-fold cross-validation. This model outperformed or matched the results of the leading single-target studies in the literature.
- The model demonstrated strong generalization capability, maintaining high predictive accuracy across all folds. This indicates robustness for industrial applications, where process variability is common.
- As the infill rate and layer thickness increase, the measured hardness values of the ASA parts gain an upward tendency, but this case is not valid for vaporizing time, and it should be optimized for target hardness levels.
- There is no direct increasing/decreasing relationship between surface roughness values and vaporizing time, so the best surface quality can be obtained with intermediate levels of acetone gas treatment duration.

Overall, this study bridges the gap between single-target research approaches and practical multi-target industrial solutions by demonstrating that CNN-enhanced ML models can accurately, reliably, and cost-effectively predict critical quality metrics in additive manufacturing. The proposed framework advances predictive modeling and offers a scalable real-world tool for accelerating process optimization, improving product consistency, and reducing manufacturing costs in high-performance engineering applications.

## Figures and Tables

**Table 1:** Significant properties of ASA filament.

Property	Outcome
Color	black
Diameter (mm)	1.75
Density (kg/m^3^)	1060
Bed Temperature (°C)	90–110
Nozzle Temperature (°C)	240–265
Material Flow (%)	100
Printing Speed (mm/s)	50–100
Fan Speed (%)	100

**Table 2:** Input variables and their levels.

Input Variable	Level
Layer thickness (mm)	0.1, 0.2, 0.4
Infill rate (%)	25, 50, 100
Vaporizing time (min)	15, 45, 90, 120

**Table 3:** Original dataset.

	Layer Thickness (mm)	Vaporizing Time (min)	Infill Rate (%)	Hardness (Shore D)	Surface Roughness (μm)		Layer Thickness (mm)	Vaporizing Time (min)	Infill Rate (%)	Hardness (Shore D)	Surface Roughness (μm)
1	0.1	15	25	59	4.02	55	0.2	90	50	43	0.53
2	0.1	15	25	60	4.89	56	0.2	90	50	45	0.69
3	0.1	15	25	61	5.12	57	0.2	90	50	46	0.52
4	0.1	45	25	64	5.28	58	0.2	120	50	39	2.19
5	0.1	45	25	65	4.33	59	0.2	120	50	43	2.22
6	0.1	45	25	65	4.56	60	0.2	120	50	45	1.87
7	0.1	90	25	47	0.34	61	0.4	15	50	54	6.19
8	0.1	90	25	46	0.46	62	0.4	15	50	55	7.39
9	0.1	90	25	48	0.56	63	0.4	15	50	54	7.77
10	0.1	120	25	43	2.12	64	0.4	45	50	58	7.95
11	0.1	120	25	42	1.17	65	0.4	45	50	56	7.83
12	0.1	120	25	43	1.44	66	0.4	45	50	59	7.91
13	0.2	15	25	56	6.23	67	0.4	90	50	41	0.72
14	0.2	15	25	56	6.34	68	0.4	90	50	44	0.79
15	0.2	15	25	55	6.92	69	0.4	90	50	40	0.94
16	0.2	45	25	59	6.34	70	0.4	120	50	41	2.05
17	0.2	45	25	58	5.78	71	0.4	120	50	40	2.28
18	0.2	45	25	59	6.18	72	0.4	120	50	38	2.72
19	0.2	90	25	42	0.61	73	0.1	15	100	72	4.83
20	0.2	90	25	43	0.54	74	0.1	15	100	70	4.37
21	0.2	90	25	43	0.49	75	0.1	15	100	73	5.81
22	0.2	120	25	39	1.82	76	0.1	45	100	75	5.64
23	0.2	120	25	40	2.06	77	0.1	45	100	74	5.76
24	0.2	120	25	38	1.67	78	0.1	45	100	78	4.45
25	0.4	15	25	53	6.76	79	0.1	90	100	59	0.38
26	0.4	15	25	52	7.02	80	0.1	90	100	58	0.41
27	0.4	15	25	51	7.25	81	0.1	90	100	62	0.53
28	0.4	45	25	55	7.19	82	0.1	120	100	54	2.49
29	0.4	45	25	58	7.63	83	0.1	120	100	56	1.57
30	0.4	45	25	57	7.96	84	0.1	120	100	58	1.66
31	0.4	90	25	40	0.81	85	0.2	15	100	60	6.75
32	0.4	90	25	39	0.85	86	0.2	15	100	63	6.97
33	0.4	90	25	41	0.98	87	0.2	15	100	61	6.44
34	0.4	120	25	38	2.67	88	0.2	45	100	69	7.14
35	0.4	120	25	36	1.94	89	0.2	45	100	67	6.74
36	0.4	120	25	36	2.83	90	0.2	45	100	69	6.58
37	0.1	15	50	64	4.34	91	0.2	90	100	49	0.46
38	0.1	15	50	65	4.96	92	0.2	90	100	53	0.62
39	0.1	15	50	62	5.68	93	0.2	90	100	54	0.55
40	0.1	45	50	69	5.45	94	0.2	120	100	45	2.44
41	0.1	45	50	69	4.76	95	0.2	120	100	48	2.64
42	0.1	45	50	66	4.31	96	0.2	120	100	50	1.77
43	0.1	90	50	50	0.27	97	0.4	15	100	57	6.23
44	0.1	90	50	51	0.38	98	0.4	15	100	56	7.86
45	0.1	90	50	53	0.44	99	0.4	15	100	59	7.17
46	0.1	120	50	50	2.36	100	0.4	45	100	62	8.15
47	0.1	120	50	51	1.35	101	0.4	45	100	66	8.13
48	0.1	120	50	51	1.59	102	0.4	45	100	61	7.96
49	0.2	15	50	56	6.48	103	0.4	90	100	45	0.81
50	0.2	15	50	57	6.93	104	0.4	90	100	47	0.86
51	0.2	15	50	54	6.88	105	0.4	90	100	44	0.92
52	0.2	45	50	63	5.94	106	0.4	120	100	42	2.56
53	0.2	45	50	61	6.88	107	0.4	120	100	39	2.33
54	0.2	45	50	60	6.77	108	0.4	120	100	37	2.64

**Table 4:** Structure and parameters of the ML and DL models.

Models	Parameters
LR	fit_intercept = 1, normalize = ‘deprecated’, n_jobs = None, positive = 0
Ridge Regression	alpha = 1.0, fit_intercept = 1, max_iter = None, tol = 0.001, solver = ‘auto’, positive = 0, random_state = None
Bayesian Ridge	alpha_1 = 10^−6^, alpha_2 = 10^−6^, lambda_1 = 10^−6^, lambda_2 = 10^−6^, fit_intercept = 1, normalize = 0
DT Regressor	random_state = 42, criterion = ‘squared_error’, max_depth = None, min_samples_split = 2, min_samples_leaf = 1
RF Regressor	n_estimators = 100, random_state = 42, criterion = ‘squared_error’, max_depth = None, min_samples_split = 2, min_samples_leaf = 1
GB Regressor	n_estimators = 100, random_state = 42, learning_rate = 0.1, max_depth = 3, min_samples_split = 2, min_samples_leaf = 1
AdaBoost Regressor	n_estimators = 100, random_state = 42, learning_rate = 1.0, loss = ‘linear’
ET Regressor	n_estimators = 100, random_state = 42, criterion = ‘squared_error’, max_depth = None, min_samples_split = 2, min_samples_leaf = 1
SVR	kernel = ‘rbf’, C = 10, gamma = ‘scale’, epsilon = 0.1
KNN Regressor	n_neighbors = 5, weights = ‘uniform’, algorithm = ‘auto’, leaf_size = 30, p = 2
XGBoost Regressor	verbosity = 0, random_state = 42, n_estimators = 100, learning_rate = 0.3, max_depth = 6, min_child_weight = 1, gamma = 0, subsample = 1, colsample_bytree = 1
Bagging Regressor	n_estimators = 50, random_state = 42, max_samples = 1.0, max_features = 1.0
Stacking Regressor	Base estimators: RF (n_estimators = 50, random_state = 42), SVR (kernel = ‘rbf’, C = 10, gamma = ‘scale’)—Final estimator: Ridge (alpha = 1.0)
MLP Regressor	hidden_layer_sizes = (100,), max_iter = 500, random_state = 42, activation = ‘relu’, solver = ‘adam’, learning_rate_init = 0.001
1D-CNN	layers = 5, units = 64, filters = 32, kernel_size = 2, activation = ‘relu’, optimizer = ‘adam’, learning_rate = 0.001, loss = ‘mse’
2D-CNN	layers = 5, units = 64, filters = 32, kernel_size = (2.1), activation = ‘relu’, optimizer = ‘adam’, learning_rate = 0.001, loss = ‘mse’
RNN	layers = 4, units = 64, rnn_units = 32, activation = ‘relu’, optimizer = ‘adam’, learning_rate = 0.001, loss = ‘mse’
LSTM	layers = 4, units = 64, lstm_units = 32, activation = ‘relu’, optimizer = ‘adam’, learning_rate = 0.001, loss = ‘mse’

**Table 5:** Structure and parameters of the feature extraction and regression heads.

Models	Parameters
1D-CNN Feature Extractor	layers = 6, units = 64, feature_units = 32, filters = 32, kernel_size = 2, activation = ‘relu’, optimizer = ‘adam’, learning_rate = 0.001, loss = ‘mse’
RF Regression Head	n_estimators = 100, random_state = 42
Ridge Regression Head	alpha = 1.0, fit_intercept = 1, max_iter = None, tol = 0.001, solver = ‘auto’
SVR Regression Head	kernel = ‘rbf’, C = 1.0, gamma = ‘scale’, epsilon = 0.1

**Table 6:** Performance results of the models.

Models	Avg MSE	MSE Std	$\mathrm{Avg}\;R^{2}$	$R^{2}$ Std
Feature Extraction with SVR	2.0941	0.4468	0.9614	0.0112
GB	2.2007	0.7024	0.9580	0.0090
RNN	2.0339	0.3210	0.9575	0.0104
1D-CNN	1.7933	0.3546	0.9571	0.0095
2D-CNN	1.9171	0.4500	0.9559	0.0093
Feature Extraction with Ridge	2.1783	0.3985	0.9556	0.0111
SVR	2.0916	0.5130	0.9547	0.0085
Bagging	2.4673	0.1428	0.9544	0.0114
Feature Extraction with RF	2.5630	0.4478	0.9540	0.0134
RF	2.5202	0.3583	0.9539	0.0123
XGBoost	2.5491	0.5790	0.9500	0.0108
Stacking	3.0780	0.5632	0.9472	0.0077
DT	2.8042	0.8436	0.9467	0.0106
ET	3.0263	1.4123	0.9446	0.0134
AdaBoost	4.7585	1.3566	0.9365	0.0171
MLP	3.5403	1.1507	0.9237	0.0064
LSTM	3.7081	2.0702	0.9048	0.0227
KNN	5.1686	1.5720	0.8964	0.0388
Ridge	13.0352	2.3789	0.6942	0.0400
Bayesian Ridge	13.0423	2.3626	0.6942	0.0395
LR	13.0641	2.3040	0.6933	0.0398

**Table 7:** Five-fold results of the top three models and the worst model.

Models	Fold 1		Fold 2		Fold 3		Fold 4		Fold 5	
	$R^{2}$ (%)	MSE	$R^{2}$ (%)	MSE	$R^{2}$ (%)	MSE	$R^{2}$ (%)	MSE	$R^{2}$ (%)	MSE
Feature Extraction with SVR	94.62	2.494	97.37	22.229	97.45	14.168	95.98	25.878	95.28	17.491
GB	94.98	1.738	97.15	21.283	96.62	20.053	95.24	35.515	95.03	15.804
RNN	94.59	17.855	97.18	19.946	96.44	19.725	95.99	26.484	94.53	17.683
LR	62.49	12.39	67.16	16.84	72.46	11.59	71.71	14.27	72.81	10.20

**Table 8:** Comparative summary of current research and related studies on predicting hardness and surface roughness in additive manufacturing.

Author(s)-Paper	Material/Process	Target Property(ies)	Model(s)	Best Performance Metrics
Hossain et al. (2022) [52]	FDM–Generic	Nozzle Temperature	ANN	$R^{2}$ = 0.985–0.996
Batu et al. (2023) [53]	Various AM	Surface Roughness	CNN, SVM, Ensemble	Accuracy = 99.2%
Veeman et al. (2023) [54]	FDM–ABS	Hardness	RF	$R^{2}$ = 0.9136
Badogu et al. (2024) [55]	FDM–PLA	Hardness	Ensemble Learning	$R^{2}$ = 0.955
Mahmoud et al. (2024) [56]	FDM–PC	Hardness	RF	$R^{2}$ = 0.995 RMSE = 0.0069
Wardhani et al. (2024) [57]	Batik Motif Dataset	Pattern Classification	CNN	Accuracy = 99.5%
Kadauw et al. (2025) [58]	SLM–Ti6Al4V	Defect Detection	CNN	Accuracy = 99.15% F1 = 0.991
Mulugumdam et al. (2025) [59]	FDM–Generic	Single Target (Quality Metric)	XGBoost	MSE = 0.799
Özkül et al. (2025) [60]	FDM–ABS	Hardness, Tensile Strength, Flexural Strength, Surface Roughness	KSTAR (hardness and roughness), MLP (tensile and flexural)	Hardness: MAE = 0.006 $R^{2}$ ≈ 0.99 Surface Roughness: MAE = 0.009 $R^{2}$ ≈ 0.99 Tensile and Flexural Strength: $R^{2}$ ≈ 0.99
Panico et al. (2025) [61]	Laser Powder Bed Fusion	Mechanical Properties	CNN-based models	$R^{2}$ ≈ 0.94
Reddy et al. (2025) [62]	FDM–ABS	Tensile Strength	XGBoost	$R^{2}$ = 0.9876 MSE = 0.214 MAE = 0.342
Tzotzis et al. (2025) [63]	CFRP Machining	Surface Roughness	ANN (Vibration Features)	MAPE = 1.51%
This study	FDM–PLA	Hardness and Surface Roughness	CNN-based Feature Extraction with SVR	$R^{2}$ = 0.9614 $R_{std}^{2}$ = 0.0112 MSE = 2.0941

## Data Availability

The data that support the findings of this study are available on request from the corresponding author. Additionally, the source code used for model development, training, and performance evaluation is openly available from the accompanying GitHub repository: https://github.com/FurkancanDemircan/ASA-Hardness-Roughness-MLDL (accessed day 18 October 2025).

## Supplementary Materials

The following supporting information can be downloaded at https://www.mdpi.com/article/10.3390/polym17212881/s1, File S1: Analysis of ISO 52900.
