# Supplementary material for: Hardness and Surface Roughness of 3D-Printed ASA Components Subjected to Acetone Vapor Treatment and Different Production Variables: A Multi-Estimation Work via Machine Learning and Deep Learning
Source: Polymers (Basel). 2025 Oct 29;17(21):2881. doi: 10.3390/polym17212881 (PMC12610749; doi:10.3390/polym17212881)
Supplement: Supplementary file 1 [file polymers-17-02881-s001.zip › polymers-3934229-supplementary.pdf]

ISO 52900 Overview

1. Introduction

Additive manufacturing (AM) represents a transformative shift in the field of production engineering. Unlike conventional subtractive or formative manufacturing methods, AM is based on the principle of building components layer by layer directly from digital models. This approach enables the fabrication of geometrically complex structures, reduces material waste, and allows for customized production at economically viable scales. With the rapid industrial adoption of AM, a clear need emerged for a unified terminology and framework to ensure consistency across design, manufacturing, and quality control processes.

To address this, the International Organization for Standardization (ISO) introduced ISO 52900, which establishes the general principles and terminology of additive manufacturing. The standard provides the foundational structure for the AM standards family (ISO/ASTM 529xx series) and serves as the baseline for understanding processes, materials, and technical definitions across the industry. By doing so, ISO 52900 facilitates communication between researchers, designers, machine manufacturers, and end-users, promoting interoperability and technological standardization on a global scale.

2. Scope and Key Concepts of ISO 52900

ISO 52900, titled "Additive Manufacturing – General Principles – Terminology", defines AM as "the process of joining materials to make parts from 3D model data, usually layer upon layer, as opposed to subtractive manufacturing methodologies." This definition clearly distinguishes AM from traditional machining and forming processes by emphasizing the additive nature of material deposition.

The standard outlines the entire process chain of additive manufacturing, which includes data preparation, material deposition, post-processing, and quality verification. The workflow typically begins with the creation of a 3D model in CAD (Computer-Aided Design) software, which is then converted to a printable file format such as STL or AMF. The digital model is subsequently sliced into thin layers and processed by the AM machine according to specified parameters such as laser power, layer thickness, and deposition speed. After printing, the part often undergoes post-processing operations, including heat treatment, surface finishing, or machining, to enhance dimensional accuracy, surface roughness, or mechanical performance.

By defining these common stages and concepts, ISO 52900 ensures that manufacturers and researchers refer to the same principles when discussing AM systems, thereby supporting global harmonization and reproducibility.

3. Classification of Additive Manufacturing Processes

ISO 52900 identifies seven primary categories of additive manufacturing processes, based on the type of feedstock material, the energy source used, and the mechanism of material consolidation. This classification forms the basis of subsequent technical standards that specify process parameters, design rules, and material compatibility.

3.1. Vat Photopolymerization

This process involves the selective curing of a liquid photopolymer resin using a light source such as a UV laser or digital projector. Techniques like Stereolithography (SLA) and Digital Light Processing (DLP) belong to this category. Vat photopolymerization provides excellent surface finish and high resolution, making it suitable for dental models, jewelry prototypes, and microfluidic devices.

3.2. Material Extrusion

In this method, thermoplastic filament is heated and extruded through a nozzle to form layers. The most common example is Fused Deposition Modeling (FDM). Due to its simplicity and cost efficiency, FDM is widely used for educational, prototyping, and low-volume production purposes. However, it generally offers lower dimensional accuracy compared to powder-based techniques.

3.3. Powder Bed Fusion

This group includes Selective Laser Melting (SLM), Direct Metal Laser Sintering (DMLS), and Selective Laser Sintering (SLS). A high-energy laser or electron beam selectively fuses or sinters fine powder particles spread across a build platform. Powder Bed Fusion (PBF) is primarily used for metals and ceramics, producing dense and mechanically robust components. It is a key technology for aerospace, medical implants, and toolmaking applications.

3.4. Binder Jetting

In this process, a liquid binding agent is selectively deposited onto a powder bed, bonding the particles to form a solid layer. Binder jetting operates at lower temperatures and enables fast production rates. It is versatile, supporting materials such as metals, ceramics, and sand. The technology is especially advantageous for casting molds and pre-sintered metal parts.

3.5. Material Jetting

Material Jetting systems operate similarly to inkjet printing, where droplets of photopolymer or wax-based material are deposited and cured layer by layer using UV light. This technique

allows multi-material and multi-color printing, which makes it ideal for aesthetic prototypes, anatomical models, and design visualization.

3.6. Sheet Lamination

Sheet Lamination involves the layer-by-layer bonding of thin sheets of material such as paper, polymer, or metal, which are subsequently cut to shape. The most recognized example is Laminated Object Manufacturing (LOM). While it offers relatively fast build times, the process is limited by its achievable resolution and material diversity.

3.7. Directed Energy Deposition

Directed Energy Deposition (DED) simultaneously feeds powder or wire material into a high-energy beam (laser, plasma, or electron beam) to melt and deposit it directly onto the substrate. Processes like Laser Metal Deposition (LMD) and Electron Beam Additive Manufacturing (EBAM) fall under this category. DED is especially useful for repairing or refurbishing metal components and fabricating large, near-net-shape parts.

4. Material Considerations in Additive Manufacturing

The performance and reliability of AM components are strongly influenced by material properties. ISO 52900 categorizes AM materials into four principal groups: polymers, metals, ceramics, and composites. Each category exhibits distinct physical and chemical behaviors, which determine process compatibility and end-use suitability.

Polymers:

Commonly used in FDM, SLA, and Material Jetting, polymers are valued for their ease of processing and cost-effectiveness. Thermoplastics such as PLA, ABS, PETG, and nylon are widely adopted, while photopolymer resins offer superior surface quality and dimensional precision.

Metals:

Metallic powders or wires are used in high-energy AM processes like SLM, DMLS, and DED. Materials such as stainless steel, titanium, aluminum, and Inconel alloys provide excellent strength-to-weight ratios, corrosion resistance, and biocompatibility. Particle size distribution, powder morphology, and oxidation level are critical parameters that influence the final part density and mechanical integrity.

Ceramics:

Although more challenging to process due to their brittleness and high melting temperatures, ceramics are suitable for binder jetting or powder-based fusion systems. They

are employed in electronics, biomedical implants, and high-temperature applications due to their thermal stability and hardness.

Composites:

Composite materials combine a matrix phase (typically polymeric or metallic) with reinforcement fibers such as carbon or glass. Fiber-reinforced polymers are increasingly utilized for lightweight structural components in automotive and defense sectors, providing high stiffness and fatigue resistance.

Material selection depends on a variety of factors, including mechanical requirements, surface finish, cost constraints, and production rate. Moreover, ISO 52900 emphasizes the importance of controlling material-related parameters—such as powder flowability, moisture sensitivity, and thermal behavior—to ensure repeatable quality and process stability.

5. Conclusion

The ISO 52900 standard serves as a cornerstone for the global additive manufacturing ecosystem by providing a common language and classification framework. Beyond terminology, it lays the groundwork for subsequent standards that define design guidelines, testing procedures, and quality assurance systems, including ISO/ASTM 52901 (Quality principles) and ISO/ASTM 52910 (Design requirements).

In the context of Industry 4.0 and digital manufacturing, ISO 52900 plays a pivotal role in ensuring interoperability among machines, materials, and software platforms. By unifying technical definitions and process categorizations, the standard enables consistency, traceability, and reliability across diverse industrial sectors.

Ultimately, ISO 52900 represents more than a terminological reference—it is a strategic framework that supports innovation, safety, and standardization in the fast-evolving field of additive manufacturing. As AM technologies continue to mature and diversify, ISO 52900 will remain essential for guiding their integration into mainstream industrial production.
